# The “Newborn Gang” Scandal in Türkiye: Ethics in a Neoliberal Health System

**DOI:** 10.1007/s10728-025-00522-5

**Published:** 2025-05-28

**Authors:** Maide Barış, Gürkan Sert, M. İnanç Özekmekçi

**Affiliations:** 1 Marmara University, Istanbul, Türkiye; 2 Erciyes University, Kayseri, Türkiye

**Keywords:** Newborn gang scandal, Medical scandal, Ethics, Neoliberalism and healthcare

## Abstract

In October 2024, Türkiye was shocked by the “Newborn Gang” scandal, in which a network of healthcare professionals allegedly exploited newborns for financial gain in private hospitals. The accused are charged with intentionally neglecting, mistreating or even killing of healthy infants in neonatal intensive care units to prolong their stays and maximize government reimbursements. This paper critically examines the structural and ethical failures exposed by the 2024 “Newborn Gang” scandal in Türkiye, in which healthcare professionals in private hospitals allegedly allowed or caused the deaths of newborns to profit from the state’s healthcare reimbursement system. Drawing on the frameworks of neoliberal critique and medical humanities, the study argues that such extreme violations are not isolated incidents of individual misconduct, but manifestations of deeper systemic vulnerabilities fostered by the neoliberalization of healthcare. It explores how deregulation, market incentives, and the erosion of ethical values—exacerbated by Türkiye’s Health Transformation Program—have created an environment where financial gain is prioritized over patient welfare. Comparative case studies are employed to contextualize these findings within broader global patterns of ethical collapse in healthcare systems influenced by market logic. The paper contends that merely strengthening oversight is insufficient; rather, a structural reorientation is needed. As a potential alternative, the study introduces Value-Based Healthcare as a model that aligns clinical outcomes with ethical imperatives. Ultimately, the paper calls for a fundamental moral recalibration of healthcare—one that affirms care, integrity, and justice as core values over profit and efficiency.

## Introduction: The “Newborn Gang” Scandal

In October 2024, a scandal known as the “Newborn Gang” in Türkiye exposed a network of healthcare professionals exploiting newborn babies for financial gain in private hospitals [3]. The gang allegedly allowed or even caused the deaths of healthy infants by intentionally neglecting or mistreating them in neonatal intensive care units (NICUs) to extend their stays and increase government reimbursements. As a result, eleven private hospitals were shut down, and 47 healthcare workers faced serious charges, including negligent homicide and fraud [14]. The gang operated by diverting newborns, often with complex health conditions, from public hospitals to private NICUs, where they were deliberately left untreated or killed to maximize profits from Türkiye’s Social Security Institution (SSI), which reimburses a daily rate for each occupied NICU bed. The investigation, which began in April 2023 following a mother’s complaint, led to multiple operations and arrests. Shockingly, it was revealed that the gang had an armed wing, including corrupt police officers, protecting their operations. The details of the indictment point to severe ethical and legal violations, including inadequate ventilation, malnutrition, and deliberate killing [17]. As more families came forward with similar experiences, public outrage grew, suggesting that the extent of the issue might be far more widespread than initially believed.

This paper argues that the neoliberalization of healthcare creates structural conditions that not only enable but also exacerbate ethical breaches, sometimes culminating in large-scale scandals, such as the Newborn Gang case. The following sections critically examine the key ethical issues at the heart of this scandal, including insufficient regulatory oversight, the marginalization of families in clinical decision-making, and the erosion of ethical values within the healthcare system. While neoliberal healthcare policies prioritize financial incentives over patient welfare, they do not directly cause extreme ethical violations like homicide. Instead, such crimes arise from the convergence of multiple systemic failures—including regulatory neglect, institutional complicity, and moral disengagement [30]. The Newborn Gang scandal illustrates how market-driven pressures create vulnerabilities that, when combined with weak oversight, can escalate into severe ethical breaches.

To support this argument, this paper draws on insights from medical humanities literature to demonstrate how neoliberal models foster professional moral decline and deepen systemic vulnerabilities in healthcare settings. Neoliberal health policies—emphasizing privatization, cost-efficiency, and profit—have been widely criticized by medical sociologists, ethicists, and philosophers for their negative impact on access to care and social justice. For instance, Michael J. Sandel, in *What Money Can’t Buy: The Moral Limits of Markets*, explores the ethical dilemmas arising when market values infiltrate traditionally non-market areas like healthcare. He argues that neoliberalism’s emphasis on deregulation and privatization has led to the commodification of healthcare, treating it as a marketable good rather than a public right. This shift erodes trust between patients and providers, prioritizing profit over patient welfare, as seen in scandals like the Newborn Gang case [32].

Similarly, in *Can Medicine Be Cured?* Seamus O’Mahony contends that neoliberalism has corrupted the medical profession. He argues that treating healthcare as a commodity incentivizes profit over patient care, leading to the erosion of professional ethics. Traditionally, doctors were dedicated to patient welfare, but market forces such as cost-cutting and profit-driven models have reshaped the profession. This transformation undermines core values like care, empathy, and public service while reinforcing a system that prioritizes financial gain. As O’Mahony suggests, this market-driven shift corrupts the ethical foundations of healthcare and erodes public trust, contributing to scandals in healthcare [28].

The Newborn Gang scandal serves as a compelling example of how market-driven healthcare policies, while not directly causing criminal behaviour, can create conditions that facilitate the exploitation of vulnerable groups. This aligns with broader concerns that such policies undermine public health and contribute to ethical lapses within healthcare systems. Analysing this scandal through the critical perspectives of Sandel and O’Mahony highlights the broader ethical costs of neoliberal healthcare systems. These include the corruption of medical professionalism, the prioritization of profit over patient care, and the increasing influence of market forces such as outsourcing, cost-cutting, and profit-driven measures. The erosion of core professional values like care and empathy creates an environment in which ethical practice becomes increasingly difficult to maintain. Within such a flawed system, ethical principles and systemic demands often conflict, creating vulnerabilities that unscrupulous individuals exploit—manipulating human lives for financial gain, a consequence that no ethical framework could justify.

The paper is structured as follows: “The Neoliberal Influence on Healthcare: The Inevitable Ethical Costs” section examines the neoliberal influence on healthcare and its ethical costs. “Ethical Breakdown of Neoliberalism Through the Lens of Scandals” section analyses these costs through case studies of health scandals in the UK and the USA. “Failure of Oversight Mechanisms: Catalyst or Consequence?” section explores whether oversight failures were a catalyst or a consequence of neoliberal healthcare policies, while “Addressing the Neoliberal Gaps in Healthcare: Individual or Systemic Actions?” section considers whether these failures stem from individual misconduct or systemic design. “Exploring Alternatives to Neoliberal-Aligned Healthcare Systems” section discusses Value-Based Healthcare (VBHC) as a potential alternative, shifting the focus from profit generation to patient-centred outcomes. Finally, “Conclusion” section concludes the paper.

## The Neoliberal Influence on Healthcare: The Inevitable Ethical Costs

To understand how such structural and ethical vulnerabilities took root, it is essential to examine the broader political and economic transformations that have shaped Türkiye’s healthcare system over the past four decades. The *Newborn Gang* scandal has drawn renewed attention to Türkiye’s increasing reliance on private hospitals and the systemic risks posed by a neoliberalized healthcare model—a concern already voiced by several Turkish scholars in the early days of the investigation [10, 29].

This paper argues that the neoliberalization of healthcare in Türkiye has contributed to financial conflicts of interest and ethical breaches by prioritizing market-driven incentives over patient welfare, as ultimately borne out by the *Newborn Gang* scandal. However, it is important to acknowledge that ethical failures in healthcare are not exclusive to neoliberal systems. Corruption, negligence, and regulatory collapse have occurred in state-controlled and mixed healthcare models as well [35]. What distinguishes neoliberal healthcare systems is not the presence of ethical failure per se, but the particular pressures they impose—pressures that embed financial logics into clinical decision-making and institutional priorities, thereby weakening the safeguards that normally uphold professional integrity.

Neoliberalism is an economic framework that seeks to optimize human welfare by fostering individual freedoms and capabilities within a system based on strong private property rights, open markets, and free trade [20]. Applied to healthcare, this model promotes a market-driven approach in which financial incentives often outweigh ethical considerations, leading to the commodification of healthcare and undermining the principle of prioritizing patient well-being. Instead, profit-maximization strategies take precedence, treating patients as consumers and healthcare professionals as service providers driven by financial incentives [20]. This profit-driven logic is further reinforced by the language used in healthcare, which increasingly reflects a business-oriented mindset rather than a patient-centred one. Terms like “customer,” “business,” and “cost-saving” have reframed healthcare as a transactional service, shifting the focus away from human values. Physicians, increasingly seen as labour providers, are now pressured to “sell diseases” rather than care for patients as whole human beings, further eroding core ethical values. Thus, it can be claimed that the terminology used in neoliberal healthcare systems not only reflects but also reinforces a transactional mindset, shaping how healthcare professionals perceive their roles and responsibilities. This shift in language can contribute to ethical erosion, as it frames patients as consumers rather than individuals in need of care and reduces physicians to service providers driven by financial incentives rather than medical duty [2].

In the early 1980s, Türkiye’s shift toward neoliberal economic policies began with the implementation of a stabilization and structural adjustment program. These policies have since reshaped nearly every aspect of economic and social life, with market-driven strategies transforming foreign trade, factor markets, agriculture, labour, and social sectors, including healthcare and education. The neoliberal Health Transformation Program (HTP), introduced in 2003, exemplifies this shift, incentivizing healthcare professionals to prioritize financial gain, often at the expense of ethical practices and equitable care. While HTP led to some improvements in healthcare delivery, such as increased access to services as well as increased efficiency in some areas, it also embedded neoliberal principles into the system, introducing significant ethical concerns [2].

HTP, like other neoliberal health regulation models globally, restructured priorities, allowing profitability to override ethical principles, such as prioritizing care based on medical need. These systemic pressures challenge medical professionalism, urging a re-evaluation of policies integrating market elements into healthcare [12]. David Harvey aptly summarizes this dynamic: “Neoliberalism produces a system where individual behaviours are shaped by systemic pressures, incentives, and constraints. It is not merely individual greed but the structural design of the system that fosters unethical practices” [20].

The *Newborn Gang* scandal starkly illustrates this logic in practice. The privatization and outsourcing of NICUs to a company run by the alleged gang leader, a paediatrician, reflects how structural weaknesses and unchecked incentives can converge. Cost-cutting strategies, such as minimizing physician oversight in neonatal care, underscore the peril of subordinating ethics to efficiency. In such an environment, “greed for profit” becomes not an exception but a normalized symptom of institutional design [2].

Given these dynamics, a critical question arises: *Can medical ethics be preserved in a commodified healthcare system driven by profit?* The answer, grounded in current systemic realities, appears to be resounding no. Healthcare professionals now operate in a framework that often conflicts with their ethical commitments. In such systems, the focus shifts from safeguarding patient welfare to maximizing revenue per case [39]. While not all harm is immediate or visible, many profit-driven decisions—such as overtreatment, neglect, or defensive medicine—can cause long-term damage, particularly in vulnerable populations.

At this point, it is essential to recognize how systemic pressures gradually normalize unethical behaviour in everyday medical practice, beyond overt ethical breaches. While the Newborn Gang scandal is an extreme case of ethical collapse, ethical erosion often unfolds gradually rather than as a single catastrophic event. Recognizing these subtler forms of ethical failure—what scholars term “slow violence”—is essential for preventing more overt and systemic ethical breaches within healthcare. As Goswami and Cherrier argue, such violence is not abrupt or spectacular, but rather accumulates gradually through routine practices shaped by institutional and ideological pressures [18]. Failing to identify and address these incremental erosions of ethical standards allows them to become normalized, potentially laying the groundwork for more significant ethical collapse. One of the most insidious ways this unfolds is through the relentless demand for efficiency. Neoliberal performance metrics such as patient quotas and pay-for-performance schemes force doctors to prioritize speed over care. Studies show that in such systems, physicians often spend less than 10 min per patient, limiting meaningful interactions and undermining trust [4]. Over time, this routinization of haste and depersonalization normalizes corner-cutting—not by individual intent, but as a systemic necessity.

Another significant driver of this ethical erosion is the commercialization of medical education. As healthcare institutions become more entangled in market logic, medical students are trained not just as future caregivers but as competitive service providers. This dual identity creates a widening gap between ethical ideals and economic imperatives, discouraging reflective ethical reasoning [4]. This shift is particularly concerning given the overwhelming evidence that financial incentives can distort medical judgment. When hospital revenue becomes the overriding priority, unnecessary tests, procedures, and prescriptions are not tolerated—they become institutional norms [31].

In this context, the *Newborn Gang* scandal should not be dismissed as an isolated incident of criminal misconduct. It represents the extreme endpoint of a broader spectrum of ethical decay made possible by systemic market forces. Ethical disengagement is not spontaneous—it is nurtured by institutional healthcare environments that reward compliance with financial incentives over professional integrity. Recognizing these subtler forms of ethical failure is crucial if we are to prevent larger ethical violations from taking root in the healthcare system.

## Ethical Breakdown of Neoliberalism Through the Lens of Scandals

To fully grasp the systemic nature of such ethical failings, it is instructive to examine similar cases from other healthcare systems where financial or professional conflicts of interest have led to serious ethical violations.

Unfortunately, several cases worldwide highlight the devastating consequences of such conflicts, echoing the underlying dynamics of the Newborn Gang Scandal. One example is the Redding Medical Center scandal (Redding, Northern California, USA) in which “a deliberate push for high profits prompted the hospital and doctors to perform inordinate numbers of highly profitable heart procedures, whether they were necessary or not” [7]. Another disturbing example is the Ian Paterson case in the UK. Over 14 years, Paterson performed unnecessary and unapproved surgical procedures on more than 1000 breast cancer patients—driven not by clinical need, but by financial incentives within a market-oriented system [24]. These cases demonstrate how profit motives, when left unchecked, can lead to gross breaches of ethical and professional standards. Another case—though more rooted in professional hierarchy than direct financial motives—is the Bristol Royal Infirmary scandal. This high-profile medical inquiry in the UK revealed widespread negligence and ethical failures in neonatal cardiac surgery. Surgeons with insufficient experience operated on high-risk infants, and the hospital failed to intervene or enforce accountability. Prestige, power consolidation, and a desire to maintain institutional viability contributed to the sustained pattern of harm [5].

While the Bristol case and the Newborn Gang scandal share similarities—such as systemic oversight failure and the exploitation of vulnerable patients—their motivations diverge. In Bristol, the primary drivers were professional ego, institutional prestige, and the suppression of dissent. By contrast, the Newborn Gang case was more explicitly motivated by financial gain, with actions including deliberate neglect and even homicide, indicating a direct commodification of human life for profit. This distinction highlights different expressions of ethical failure within structurally weakened systems. Despite these contextual differences, Türkiye’s and the UK’s healthcare systems share important structural similarities. Both are publicly funded, single-payer systems designed to provide universal healthcare. As such, comparing the Bristol and Newborn Gang cases is both appropriate and revealing, as both involve institutional tolerance for unethical practices that exploited vulnerable patients [13]. In the UK, the Bristol case prompted public outcry and significant institutional reform [1, 33]. Similarly, the Newborn Gang scandal exposes a corruption of conscience enabled by systemic financial conflicts of interest—a crisis that demands urgent ethical, legal, and policy-based responses. As the perpetrators of the Newborn Gang stand trial (as of December 2024), the case continues to reveal the depth of systemic breakdown in Türkiye’s healthcare system. It serves as a stark reminder of how intertwined personal gain, institutional inaction, and weak oversight can result in widespread harm.

When the reactions to the Bristol case and the proposed actions in response were examined, several parallels can be identified regarding the causes and implications of Bristol and Newborn Gang tragedies. Both cases share key ethical concerns, such as inadequate oversight, the marginalization of families in decision-making, and the decay of ethics in healthcare. Beyond the individual actions of perpetrators, three systemic ethical failures emerge from Newborn Gang case:

*Inadequate Oversight and Regulatory Failure:* the scandal reveals clear signs of inadequate or ineffective inspections [14]. In Türkiye, as in many other countries, maternal and infant deaths are subject to official scrutiny. In principle, when a newborn dies, the family may request an autopsy if they suspect foul play, or they may choose to bury the child without further investigation. However, even if a family does not have a suspect and decides to bury their child, the case is not closed. The file of the deceased infant is forwarded to the Mother and Infant Mortality Commission at the Provincial Directorate of Health, where it is reviewed to determine whether the death was natural or unnatural [25]. Thus, while maternal and infant mortality are usually areas of scrutiny, the longevity of this criminal network over nearly a decade suggests that the existing mechanisms for reporting, reviewing, and responding to these deaths were either ineffective or ignored.

*Marginalization of Families in Decision-Making:* testimonies from grieving families reveal how they were excluded from critical decisions and left uninformed about the treatment of their newborns [17]. Families were not treated as partners in care but as passive bystanders, stripped of their right to information and transparency. This systematic disregard for family involvement constitutes a major ethical violation, undermining trust and accountability in the physician–patient-family relationship.

*Erosion of Ethical Culture in Healthcare Institutions:* the scandal underscores a deeper erosion of ethical values and principles among healthcare professionals and more alarmingly, within the institutional culture itself. The behaviour exhibited by certain healthcare professionals, as members of this criminal group, represents a stark violation of the core principles that guide the healthcare profession—principles such as safeguarding health, preserving life, avoiding deliberate harm, and prioritizing the well-being of patients above personal interests. However, this isolated incident should not be used to fuel prejudice against doctors and nurses in Türkiye, as it represents the criminal actions of a small group and does not reflect the dedication of the majority of healthcare professionals.

Ultimately, this scandal cannot be viewed as a collection of individual transgressions. It reflects a broader moral and structural collapse that has been enabled by the neoliberal transformation of healthcare. This transformation has reoriented priorities away from patient welfare toward institutional survival and profitability. As such, addressing the ethical issues revealed by the Newborn Gang scandal requires not only legal justice, but also systemic reform—in oversight, medical ethics education, healthcare financing, and institutional accountability.

## Failure of Oversight Mechanisms: Catalyst or Consequence?

Among the ethical failures outlined above, the breakdown of oversight mechanisms warrants deeper scrutiny—both as an institutional failure and a symptom of systemic moral decay. The collapse of these mechanisms—both at the ministerial and hospital levels—played a crucial role in enabling unethical practices to persist unchecked, ultimately culminating in criminal acts, as seen in the *Newborn Gang* scandal. However, this failure was not merely a cause but also a consequence of the neoliberal HTP policies, which weakened regulatory capacity while incentivizing profit-maximization over patient care. Without effective external oversight and internal accountability, the financial conflicts of interest embedded in neoliberal healthcare models were further exacerbated. As misconduct became normalized, the lack of intervention further degraded institutional culture. This cyclical failure—where weak oversight enables violations and those violations, in turn deepen oversight function—reveals a core structural vulnerability within the Turkish healthcare system.

At the institutional level, regulatory bodies such as the Social Security Institution (SSI) and the Ministry of Health failed in their ethical duty to ensure compliance and protect vulnerable patients. The reliance on financial reimbursement models without sufficient auditing mechanisms allowed NICUs to exploit gaps in oversight for profit. That the gang’s operations persisted for at least 8 years—since the first reported case in 2016—strongly suggests institutional negligence. Some members of the Maternal and Infant Mortality Commission, along with forensic experts, may have acted in ways that directly or indirectly concealed unethical deaths [17]. Whether through complicity or inaction, these failures reflect not just bureaucratic inefficiency but an ethical breach of duty, as they allowed economic interests to supersede the protection of vulnerable newborns. In this context, regulatory failure must be understood not simply as a procedural lapse, but as an ethical failure in its own right. The inability—or unwillingness—of institutions to detect fraudulent claims, investigate malpractice, or challenge unethical behaviour represents a collapse of the ethical responsibilities entrusted to public health authorities.

At the hospital level, internal oversight was equally absent or compromised. Rather than upholding professional integrity, hospital administrators either ignored warning signs or were complicit in fraudulent practices. The scale of this scandal indicates a system-wide erosion of ethical standards, particularly given that the NICUs involved were externally operated by a company owned by the gang’s alleged leader—a licensed paediatrician. The privatization and outsourcing of NICUs, a clear example of a neoliberal restructuring of the healthcare system, blurred lines of responsibility, making institutional accountability even harder to enforce. Doctors charged in the case are accused of falsifying medical records and billing for unnecessary treatments and drugs to exploit the Social Security Institution (SSI) for profit. Many were routinely absent when the babies died, as the gang’s model minimized their involvement to reduce labour costs through delegating care to understaffed and undersupervised personnel. Nurses—often working alone—provided neonatal care with minimal supervision, as doctors were engaged for only a few hours a day [17]. And, the lack of independent ethics committees and patient advocacy mechanisms further worsened this environment, effectively removing all internal checks against misconduct. In such a setting, profit-centred logic overrode ethical judgment, as professionals faced minimal internal resistance when prioritizing revenue over care.

As David Harvey highlights, neoliberalism centres on market exchange as a primary motivation, often leading to reduced oversight in healthcare. This shift prioritizes profit-driven models over regulatory protections, enabling cost-cutting measures that weaken labour rights and ethical standards [20]. As a result, healthcare institutions may increasingly function under competitive pressures that devalue patient care and workplace well-being, fostering environments where incivility, exploitation, and ethical breaches become more prevalent. Similarly, Whyte examines how market-driven motives in neoliberalism transform moral frameworks, suppressing regulatory oversight, and fostering ethical breaches. She argues that neoliberalism reshapes human rights discourse by embedding market logic into moral principles, redefining rights in ways that prioritize economic efficiency over social justice [38]. This transformation weakens state intervention and regulatory mechanisms, framing moral responsibility through competition and individual market participation rather than collective welfare.

At this point, it is important to determine whether the lack of oversight is an ethical problem in itself or primarily a driver of unethical consequences. In this case, the evidence strongly supports the latter. Oversight failure is not merely a procedural issue but an ethical one, as it reflects institutional disregard for accountability and the protection of vulnerable groups. This lack of oversight—both at the institutional (ministry) level and within the internal systems of the hospitals involved—creates an environment where unethical practices can thrive. The absence of proper supervision and governance fosters a culture of negligence, enabling ethical lapses and facilitating the exploitation of vulnerable individuals. Therefore, oversight failure is not only an ethical problem in its own right but also a catalyst for the broader ethical violations seen in this scandal.

Consequently, it is critical to note that the presence or absence of oversight mechanisms cannot rebuild ethics in a market-driven system, as market logic perpetuates financial conflicts of interest at every level. Unethical behaviour cannot be understood outside the systemic conditions that foster it. The Newborn Gang scandal, therefore, goes beyond regulatory failures; it reflects a profound decay in personal and professional ethics, driven by systemic flaws that breed financial conflicts of interest. The persistence of the gang’s operations, resulting in the deaths of vulnerable infants, underscores the depth of moral collapse at the systemic level, far beyond individual or regulatory shortcomings.

## Addressing the Neoliberal Gaps in Healthcare: Individual or Systemic Actions?

Building on the ethical failures discussed above, it is crucial to examine whether these failures stem primarily from individual misconduct or systemic design. This question lies at the heart of both the Bristol and Newborn Gang scandals, as it determines the direction of reform: should we focus on individual accountability, or must we challenge the system itself?

Following the Bristol case, several authors insightfully pointed out that the culture that can lead to devastating outcomes of the Bristol case must be understood in order to enhance the situation [1, 26, 27]. Was this culture fermented within the healthcare profession due to individual misconduct or systemic failures?

The answer for this question was important because according to the diagnosis of the pathology, actions can follow two main different directions. The first one is the “person approach” in which individual behaviour of the healthcare professionals should be focused and enhanced. The second one is the “system approach” in which the systemic influences which predispose healthcare professionals to behave in this way should be focused and managed [30]. For the former direction, The Code of Conduct for NHS Managers was published in response to the Bristol Report [11]. However, according to some authors, “None of these duties have impinged much on professional behaviour” [27].

Several scholars have concluded that the root cause of the Bristol case lay not in individual wrongdoing but in system-level failures that created the conditions for unethical behaviour [37]. As Smith warned:

If the Bristol case leads to an environment where we concentrate on removing bad apples rather than improving the whole system then both patients and doctors will suffer. There must be mechanisms for responding to doctors whose performance has deteriorated to an unacceptable level, but such mechanisms will never bring about the systemic improvements that we need [33]

In a similar vein, right after the Newborn Gang scandal was revealed, Civaneri stated that “The problem is not ‘gangs’ or ‘rotten apples in the basket’; the real source of the problem is the basket that rotted those apples!” [8]. This paper aligns with that perspective. It contends that the Newborn Gang scandal cannot be meaningfully understood without acknowledging the role of Türkiye’s neoliberal HTP in shaping healthcare practices over the past two decades.

However, one might ask, “Could the Bristol and Newborn Gang scandals have been prevented with more rigorous inspections?” It’s essential to consider whether improving inspections alone would address such deep-rooted issues. Crises in neoliberal healthcare systems are often reduced to simplistic causes like “insufficient oversight” or “individual malice,” but such analyses overlook the systemic roots tied to neoliberalism’s destructive effects. Neoliberal healthcare reforms prioritize market performance and economic efficiency, often at the expense of ethical safeguards. The problem seems not to lie in a lack of oversight, but in a system that commodifies oversight itself. The ineffectiveness of oversight mechanisms is an inevitable consequence of a system that views regulation as a “bureaucratic burden” or “loss of efficiency” whenever it interferes with market operations. Therefore, failures in oversight or individual misconduct are symptoms of deeper structural issues. When a system incentivizes unethical behaviour through market pressures, individual immorality becomes a predictable outcome [34]. This issue cannot be fixed by improving oversight alone; the incentive structures at the system’s core must be re-examined. Behaviours seen as immoral at the individual level are directly linked to a system that pushes individuals to make market-driven decisions. In the Newborn Gang scandal, healthcare workers’ unethical actions for profit resulted from the neoliberal system’s reward structure. This is less about individual morality (i.e., “rotten apples”) and more about the market logic embedded in the system (i.e., “the basket”). Explaining scandals purely in terms of individual immorality overlooks the broader impact of market values on medical ethics.

This argument is supported by James Reason’s Swiss Cheese Model, a widely recognized framework used to explain organizational accidents or failures, which illustrates how accidents or systemic breakdowns typically result from multiple layers of weaknesses rather than a single cause. To simply visualize this model, imagine a series of layers, each representing a safeguard, procedure, or control designed to prevent accidents or failures. These could be physical safety measures (e.g., barriers, alarms) or procedural safeguards (e.g., protocols, checklists). Each layer of defence is like a slice of Swiss cheese, with holes in it. These holes represent weaknesses or vulnerabilities in the system—places where safeguards are inadequate, ignored, or bypassed. These holes could be due to various factors, such as human error, technical malfunctions, or poor systemic design. Under normal circumstances, these layers of defence work together to prevent accidents. However, an accident occurs when the holes in different layers align. This alignment creates a path that allows hazards to reach their target (e.g., a patient or a machine). Under neoliberal healthcare systems, these “holes” expand due to market pressures that prioritize profit over patient welfare. Policies driven by cost-efficiency and market logic create systemic weaknesses, such as reduced oversight, underfunded public health mechanisms, and financial conflicts of interest. In the context of the Newborn Gang scandal, this alignment of vulnerabilities demonstrates how systemic flaws created an environment ripe for ethical violations [30].

In other words, unethical practices are predictable outcomes in a system that commodifies healthcare. The Newborn Gang scandal exemplifies how systemic failures can align to produce devastating consequences. Market-driven incentives encourage unethical actions, such as falsifying medical records and billing for unnecessary treatments, creating a cascade of failures at institutional and individual levels. Thus, while neoliberal reforms alone do not directly cause extreme ethical violations, they create a permissive environment where a lack of regulatory control, institutional alignment with profit motives, and weakened moral frameworks in healthcare can allow such failings to emerge.

Consequently, addressing such deep-rooted failures requires more than improved oversight. It demands a re-evaluation of the values that guide healthcare systems. Health policies are not just technical matters, they are moral choices. Whether a system prioritizes profit, or patient welfare will shape not only how care is delivered, but whether ethical conduct can survive.

## Exploring Alternatives to Neoliberal-Aligned Healthcare Systems

If systemic failures made unethical behaviour possible, then rebuilding ethical foundations requires not only stronger oversight, but also a fundamental rethinking of how healthcare systems are structured and valued. Addressing the roots of crises like the Newborn Gang scandal calls for more than surface-level reform—it requires a moral and institutional recalibration that prioritizes patient welfare over profit.

Medical sociologist Catherine Walsh critiques neoliberal policies for their impact on healthcare professionals, particularly physicians and nurses [36]. She argues that the emphasis on cost-efficiency and productivity often leads to burnout and job dissatisfaction, ultimately undermining the physician–patient relationship by transforming healthcare into a transactional interaction rather than a therapeutic partnership based on trust and care. The Newborn Gang scandal exemplifies the challenges Walsh identifies: systemic pressures that prioritize financial gain over ethical practice create an environment ripe for unethical behaviour.

Moreover, the involvement of healthcare workers in such crimes has also eroded public trust—both in individual providers and in the healthcare system as a whole. Trust operates in healthcare functions on two interconnected levels: between individual patients and doctors, and between society and healthcare institutions. Unfortunately, this scandal undermines both dimensions. The most profound consequence, though difficult to measure, may be the lasting damage to the doctor-patient relationship, which hinges on empathy, honesty and care.

To restore trust, healthcare systems must confront some difficult questions: how can cognitive strategies of ethical disengagement be dismantled? How can incentives structures be aligned toward ethical goals? How can opportunities for misconduct be reduced in the everyday life of hospitals?

Exploring these questions requires engaging with alternative healthcare models that balance financial sustainability with ethical integrity and rethinking how healthcare systems balance financial sustainability with ethical integrity. One such model is Value-Based Healthcare (VBHC), a framework that shifts emphasis from profit generation to patient-centred outcomes. VBHC prioritizes quality of care over quantity of services, aiming to reward effectiveness rather than volume. According to the European Commission’s Expert Panel on Effective Ways of Investing in Health [15], VBHC is grounded in four interrelated dimensions of value: allocative value (equitable distribution of resources), technical value (maximizing outcomes per resource used), personal value (care that aligns with individual patient goals), and societal value (contribution to broader social well-being). This multidimensional understanding of value reflects a shift toward a more holistic, equitable, and outcome-oriented health system. Regarding the first question, VBHC suggests that addressing cognitive strategies of ethical disengagement in the hospital setting requires a comprehensive approach that tackles both individual and systemic factors. One key strategy is promoting self-awareness and reflection among healthcare professionals through regular ethics discussions, case studies, and reflective practices. Studies indicate that physicians who receive continuous ethics training are more likely to uphold professional values and resist financial or institutional pressures that conflict with patient welfare [21]. Ethics education also helps build attributes such as honesty, empathy, and professional responsibility [6], which are essential for restoring credibility to the medical profession.

Moreover, recognizing and rewarding ethical behaviour, patient-centred care, and collaboration can help cultivate a culture where integrity and compassion are valued over financial gain or efficiency. Hospitals and healthcare organizations can introduce clear ethical guidelines and standards, linking performance evaluations and promotions to adherence to these principles. For example, rewarding teamwork, patient advocacy, and transparent communication with patients and families can encourage healthcare workers to prioritize patient well-being over external financial pressures [15]. By instilling a deeper understanding of care and hospitality, healthcare professionals can be better equipped to recognize and counteract the normalized harmful practices that perpetuate this violence.

In addition to integrating ethics education, to prevent ethical failures like those seen in the *Newborn Gang* case, it is essential to integrate structured ethical deliberation into healthcare institutions. Diego Gracia’s framework for ethical case deliberation emphasizes the importance of collective reflection in morally complex situations, ensuring that ethical concerns are not sidelined in favour of financial or institutional pressures [19]. In the Newborn Gang scandal, the lack of open ethical discussion allowed misconduct to persist until it escalated into systemic abuse. Establishing ethics committees that engage healthcare professionals in regular deliberation could serve as a safeguard against such failures. These committees would provide spaces for discussing difficult cases, reinforcing moral responsibility, and ensuring that ethical considerations are actively integrated into medical decision-making. Rather than relying solely on legal intervention after misconduct has occurred, ethical deliberation fosters a proactive culture of accountability and integrity, helping to prevent unethical practices before they become ingrained in the system.

Similarly, Cortina, through the concept of *civil ethics*, argues that ethics should not be dictated solely by law but must also be actively cultivated within institutions and society [9]. Preventing similar scandals requires more than legal prosecution; it necessitates fostering an ethical hospital culture through policies, leadership, and education that embed ethical values rather than merely enforcing legal mandates.

Per the second question, VBHC suggests changing incentives in the healthcare system to promote ethical behavior and prioritize patient welfare over profit requires a multifaceted approach that addresses both financial and non-financial motivators. Unlike market-driven systems that prioritize revenue generation, VBHC seeks to improve healthcare quality by aligning provider incentives with long-term patient well-being. In this model, healthcare providers are rewarded based on the effectiveness of treatments and overall health improvements rather than the volume of services delivered [16]. Shifting toward value-based care reduces unnecessary medical interventions, promotes shared decision-making between doctors and patients, and ensures that financial motivations do not overshadow ethical principles [22].

Regarding the third question, VBHC emphasizes transparency, accountability, and ethical decision-making to decrease opportunities for misconduct in hospitals. First, strengthening oversight and accountability is crucial. This includes conducting regular internal and external audits, establishing clear reporting mechanisms, and ensuring accountability at all levels, from clinical staff to hospital leadership [23]. When ethical deficiencies go unaddressed, they corrode trust in healthcare institutions and contribute to systemic misconduct. In the *Newborn Gang* case, failures in ethical governance allowed misconduct to persist until it escalated into outright crime. The adoption of VBHC in Türkiye could mitigate many of the ethical concerns highlighted in the Newborn Gang scandal by reducing profit-driven decision-making and reinforcing patient-centred care.

## Conclusion

This paper argues that the neoliberalization of healthcare creates structural conditions that exacerbate ethical breaches, as exemplified by the Newborn Gang scandal, which shows how market-driven pressures and weak oversight can lead to severe ethical violations or even criminal acts.

At this point, this scandal must serve as a catalyst for addressing the deep failures within the healthcare system. The fact that babies were allowed to die for profit exemplifies the urgent need to reform financial incentive structures in healthcare. These reforms must focus on preventing such atrocities from occurring in the first place, rather than merely punishing individuals after the fact.

The unethical practices exposed in this scandal, which undermine the credibility of medicine and healthcare professionals, have far-reaching consequences that threaten the very foundation of trust between healthcare providers and patients. A holistic, ethics-driven approach to healthcare, such as value-based healthcare (VBHC) model, is required to reconcile the incompatibilities between the neoliberal healthcare agenda and medical ethics. Key reforms should include empowering core ethical education in healthcare, reducing the influence of market forces, and enforcing stronger accountability mechanisms in private healthcare.

## Data Availability

No datasets were generated or analysed during the current study.
